# Origin of the Failure of Density Functional Theories in Predicting Inverted Singlet–Triplet Gaps

**DOI:** 10.1021/acs.jpca.1c10492

**Published:** 2022-02-11

**Authors:** Soumen Ghosh, Kalishankar Bhattacharyya

**Affiliations:** Max-Planck-Institut für Kohlenforschung, Mülheim an der Ruhr D45470, Germany

## Abstract

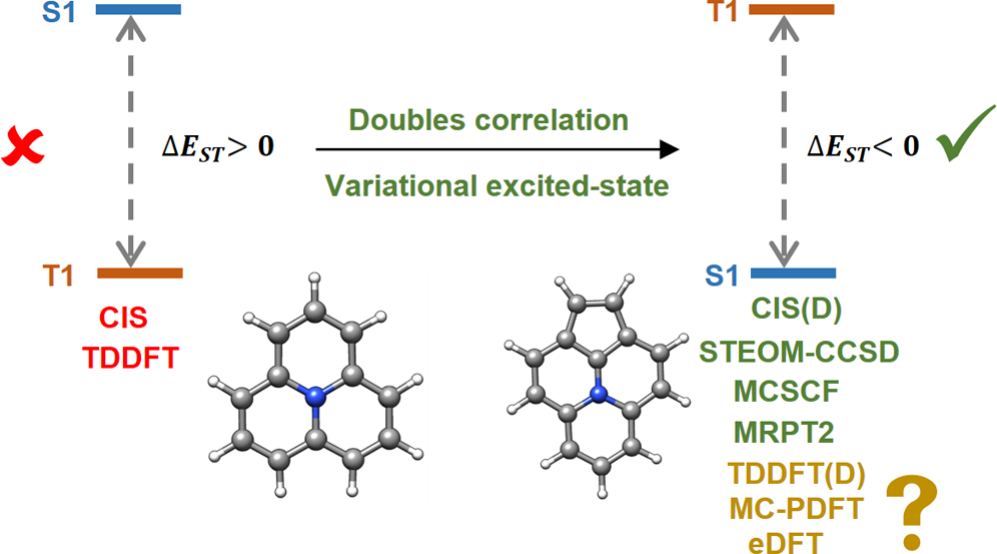

Recent experimental and theoretical studies have shown several new organic molecules that violate Hund’s rule and have the first singlet excited state lower in energy than the first triplet excited state. While many correlated single reference wave function methods have successfully predicted excited-state energetics of these low-lying states, conventional linear-response time-dependent density functional theory (TDDFT) fails to predict the correct excited-state energy ordering. In this article, we have explored the performance of combined DFT and wave function methods like doubles-corrected TDDFT and multiconfiguration pair-density functional theory for the calculation of inverted singlet–triplet gaps. We have also tested the performance of the excited-state DFT (eDFT) method for this problem. Our results have shown that it is possible to obtain inverted singlet–triplet gaps both by using doubles-corrected TDDFT with a proper choice of double-hybrid functionals or by using eDFT.

## 1 Introduction

Ground-state electronic structures and low-lying singlet and triplet excited states play important roles in organic electronic materials.^1−6^ In organic light-emitting diodes (OLEDs), charge recombination is an important step that produces singlet or triplet excitons from spatially separated holes and electrons. Generation of photons from singlet excitons happens through a spin-allowed de-excitation process. However, due to the spin forbidden nature of the de-excitation process of triplet excitons, these often contribute to energy loss. Several strategies have been developed for transforming triplet excitons produced during the recombination process to singlet excitons. In many OLEDs, triplet state (T_1_) to singlet state (S_1_) reverse intersystem crossing (RISC) is achieved by tuning the energy gap (Δ*E*_ST_) between energetically close S_1_ and T_1_ excited states. The process of RISC followed by de-excitation is referred to as thermally activated delayed fluorescence (TADF).^7−9^ Both experiments and computational modeling have contributed significantly in finding efficient design principles for TADF materials.^10−17^ Most TADF materials developed so far have small but positive Δ*E*_ST_ gaps as Hund’s rule^18^ predicts that the first excited state of a closed-shell molecule is a T_1_ state, and the S_1_ excited state will always be higher in energy. However, in the past, some N-doped triangle-shaped molecules have been suspected to have near degenerate or even inverted singlet–triplet gaps (Δ*E*_ST_ < 0).^19,20^ These types of molecules can benefit from the efficient RISC process from T_1_ to S_1_ state, leading to substantial fluorescence rates; thus, in turn, they are suitable candidates for TADF materials.

**Figure 1:**
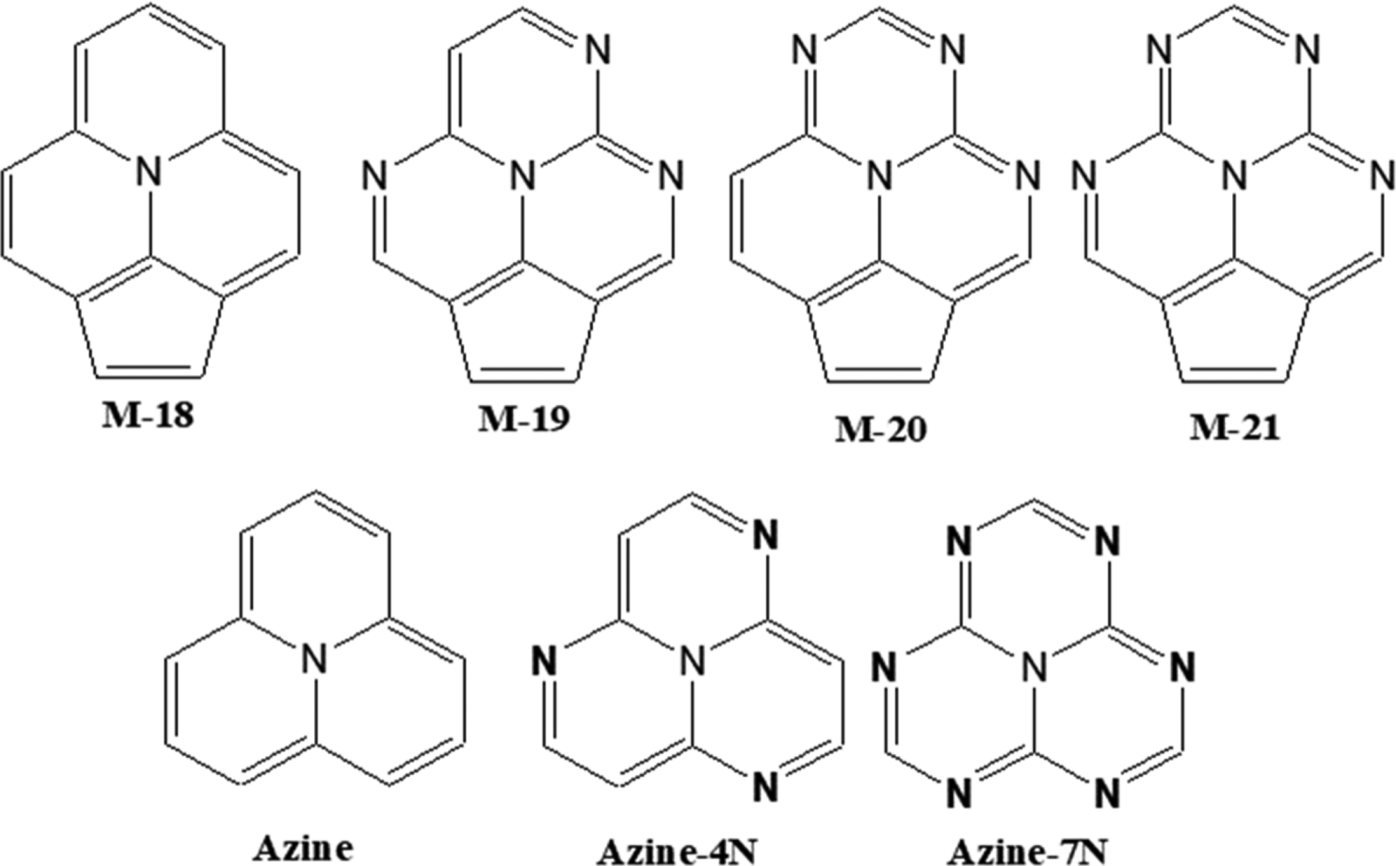
Chemical structures of molecules investigated in this work. Hydrogen atoms are not drawn here for clarity.

In recent years, Domcke and co-workers first identified heptazine derivatives to have inverted singlet–triplet gap using high-level electronic structure methods.^13^ Later, Domcke and co-workers studied electronic structure and optical properties of different azine and heptazine derivatives.^15,16^ de Silva also theoretically established existence of molecules with inverted singlet–triplet gaps using wave function methods like doubles-corrected configuration interaction singles [CIS(D)],^21^ algebraic diagrammatic construction (ADC), and equation of motion coupled cluster singles and doubles (EOM-CCSD)^33^ and also identified inability of linear-response time-dependent density functional theory (LR-TDDFT)^22^ to compute inverted singlet–triplet gaps.^14^ Dinkelbach et al. studied the effect of negative singlet–triplet gap and vibronic coupling in heptazine derivatives using the DFT/multireference configuration interaction (DFT/MRCI) approach.^23^ Sancho-Garcia and co-workers suggested that small chemical modifications of the triangle derivatives can produce good candidates for inverted singlet–triplet gap using several single reference correlated methods like spin-component scaled second-order coupled cluster (SCS-CC2),^24^ second-order ADC [ADC(2)],^25^ and multireference methods like complete active space self-consistent field (CASSCF) and N-electron valence second-order perturbation theory (NEVPT2).^26−28^ Domain-based local pair natural orbital (DLPNO) similarity transformed EOM-CCSD (STEOM-CCSD)^29^ have accurately predicated inverted singlet–triplet gaps as well.^30^ Recently, Miyajima et al. experimentally confirmed inverted singlet–triplet gaps in heptazine derivatives.^31^ While many correlated wave function-based electronic structure methods have predicted inverted singlet–triplet gaps qualitatively correctly, one of the most widely used excited-state electronic structure method, LR-TDDFT, has failed to predict the inversion of singlet–triplet gaps. Computational studies, in the past, pointed toward the inability of LR-TDDFT to incorporate double excitations by going beyond adiabatic approximation^32^ as the source of this error.^14^ Pollice et al. identified computationally a set of organic chromophores that showed efficient TADF processes using the doubles-corrected TDDFT [TDDFT(D)) method with ωB2PLYP double-hybrid functionals.^15^ While TDDFT(D) with ωB2PLYP did not always provide proper inverted singlet–triplet gaps, Pollice et al. benchmarked the method using the coupled-cluster single and double (EOM-CCSD)^33^ method to get an estimation of the systematic error. However, effect of the choice of different double-hybrid functionals on the performance of the TDDFT(D) method has not been investigated yet. Like the TDDFT(D) method, there are other combined wave function and density functional methods that have potential to be successful for inverted singlet–triplet systems.^34^ In this regard, we have examined the accuracy of a combined wave function and density functional method, known as multiconfiguration pair-density functional theory (MC-PDFT)^34,35^ in predicting energetics of low-lying singlet and triplet excited states of seven test systems (Figure 1). We have also examined the accuracy of single reference correlated wave function methods with respect to multireference second-order perturbation theory (MRPT2).

It is also possible to calculate excitation energies within the KS-DFT framework using time-independent approaches.^36^ It has been shown previously that variational optimization of the excited state within unrestricted DFT framework can provide accurate inverted singlet–triplet gaps,^13^ but such an approach can only be applied if ground and first singlet excited states are of different symmetry. More general approach is needed for variational optimization of arbitrary excited-state densities within DFT framework without any symmetry constrain. The ΔSCF method is one of the earliest time-independent DFT methods for excited states and also referred to as the excited-state DFT (eDFT) method.^37,38^ In the eDFT method, a non-Aufbau occupation of KS orbitals is imposed during self-consistent field procedure to converge the KS solution to an excited state. In the past, eDFT has been used as an alternative to TDDFT in many cases.^39−42^ In this article, we have tested the performance of the eDFT method for calculating inverted singlet–triplet gaps.

## 2 Computational Details

All LR-TDDFT, LR-TDDFT(D), CIS, random-phase approximation (RPA), CIS(D), doubles-corrected RPA [RPA(D)], and DLPNO–STEOM–CCSD calculations were performed using a development version of ORCA 5.0^43^ software. All restricted active space self-consistent field (RASSCF),^44^ RASPT2,^45^ and MC-PDFT calculations were performed using OpenMolcas software package^46^ (v19.11, tag 1689-g1367d6fd9). All RASPT2 calculations used an imaginary shift of 5.44 eV to alleviate intruder states.^47^ All calculations used Alrich’s def2 triple zeta with polarization basis functions, def2-TZVP.^48^ Multireference calculations on azine-4N, azine-7N, M-20, and M-21 were performed using *C*_*s*_ symmetry. Multireference calculations on azine and M-18 were performed using *C*_1_ and *C*_2*v*_ symmetry, respectively. Geometries of molecules in the test set were optimized in the gas phase at the B3LYP^49^/def2-TZVP level of theory, including Grimme’s D3 dispersion correction^37^ and the Becke-Johnson damping function^38^ using ORCA software. Vibrational frequency analyses were performed on all optimized geometries to confirm their nature as local minima. The eDFT calculations are performed using NWChem software package.^50^ For the eDFT calculation in NWChem, a modified SCF procedure is used where lowest (*N* – 1) orbitals and (*N* + 1)th orbital were occupied at each density matrix update step (*N* = number of occupied orbitals in the ground state). In cases where variational collapse to the ground state has occurred, the maximum overlap approach is used along with the eDFT approach to converge the SCF solutions to correct excited states.

### 2.1 Choice of Active Spaces

RASSCF calculations were performed for all seven molecules using full π-valence active spaces. In the RASSCF formalism, active space of a system can be divided into three subspaces—RAS1, RAS2, and RAS3. A full-CI calculation is performed within the RAS1 subspace. RAS2 always contains doubly occupied orbitals, and RAS3 contains unoccupied orbitals. Excitation allowed from the RAS1 subspace to other subspaces can be controlled by mentioning maximum number of holes allowed in the RAS1 subspace. Similarly, maximum number excitations into the RAS3 subspace can be controlled by mentioning maximum number of electrons allowed in the RAS3 subspace. For all the molecules in our test case, minimal active space is chosen for RAS2. That means when HOMO and HOMO-1 are degenerate for a molecule, four electrons and four orbitals are included in the RAS2 subspace, and in other case, two electrons and two orbitals are included in the RAS2 subspace. Rest of the occupied π orbitals are included in the RAS1 subspace, and unoccupied π* orbitals are included in the RAS3 space. For azine, azine-4N, and azine-7N, RAS2 only includes HOMO and LUMO, RAS1 includes six occupied orbitals, RAS3 includes six unoccupied orbitals, and maximum two holes and two electrons are allowed in RAS1 and RAS3, respectively. For RASSCF calculations on M-18, M-19, M-20, and M-21, RAS2 includes HOMO, HOMO-1, LUMO, and LUMO+1, RAS1 includes six occupied orbitals, RAS3 includes six unoccupied orbitals, and maximum two holes and two electrons are allowed in RAS1 and RAS3, respectively.

## 3 Results and Discussion

Herein, we have examined electronic structures of ground, S_1_ and T_1_ states of seven molecules, as shown in Figure 1. Among the seven molecules, azine and azine-4N are among the earliest discovered molecules that showed inverted singlet–triplet gaps. Singlet–triplet gaps for azine and azine-4N were measured to be ∼−0.08 and −0.16 eV, respectively, from the experiment.^19,20^ Recent theoretical studies have confirmed that these two molecules indeed have inverted singlet–triplet gaps.^14,17,30^ Azine-7N or heptazine have been well explored in recent years for its inverted singlet–triplet gap property. Previous theoretical calculations have predicted that azine-7N has an inverted singlet–triplet gap of ∼−0.25 eV.^12^ M-18, M-19, M-20, and M-21 molecules were identified as inverted singlet–triplet gap systems after a massive computational screening performed by Pollice et al.^17^ Pollice et al. predicted these four molecules to have promising blue emitting properties.^17^

### 3.1 Performance of TDDFT and TDDFT(D)

Within the LR-TDDFT regime, excitation energies can be calculated from the solution of the non-Hermitian eigenvalue problem
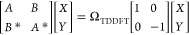
1where *X* and *Y* are excitation and de-excitation amplitudes, respectively, and Ω_TDDFT_ is the excitation energy matrix. Matrix elements corresponding to *A* and *B* matrices for a general hybrid exchange–correlation functional can be expressed as
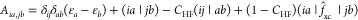
2
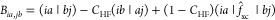
3where *C*_HF_ is the percentage of Hartree-Fock (HF) exchange in the exchange–correlation functional, (*ia*|*jb*) represents the exchange integral ∬φ_i_^*^φ_*a*_1/|*r* – *r*^′^|φ_j_^*^φ_*b*_, (*ij*|*ab*) represents a Coulombic integral, *f̂*_xc_ is the exchange–correlation kernel, and ε is the energy eigenvalues. In the case of RPA or time-dependent HF, *C*_HF_ is equal to 1, and the exchange–correlation kernel is zero. Head-Gordon and co-workers developed the CIS(D) method to account for correlation energy resulting from double excitations in a perturbative way.^21^ Neese and Grimme extended this approach to LR-TDDFT for double-hybrid exchange–correlation functionals following the same approach as CIS(D).^51^ Ottochian et al. also reported an implementation of TDDFT(D) method.^52^ In the case of double-hybrid functional, exchange–correlation energy is given by
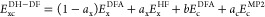
4where *a*_x_ is the HF exchange scaling parameter, *b* and *a*_c_ scale the density functional correlation and perturbative correlation contributions, respectively, *E*_x_^DFA^ and *E*_x_^HF^ are the local density functional and HF exchange, respectively, *E*_c_^DFA^ is the local density functional correlation, and *E*_c_^MP2^ is the nonlocal correlation. In case of hybrid functionals, *a*_c_ is equal to zero.

Development of the CIS(D) and TDDFT(D) method is based on the assumption that SCF and PT2 contributions to the excitation energy are additive in nature. Excitation energies in the LR-TDDFT(D) method is as follows
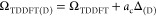
5where Ω_TDDFT_ is the excitation energy obtained from TDDFT, Δ_(D)_ is the perturbative doubles correction obtained from PT2, and *a*_c_ is the scaling factor for PT2 correlation in double-hybrid functionals (eq 4). Scaling factor, *a*_c_, is equal to 1 for CIS(D) and RPA(D) methods. Within TDDFT and CIS methods, the B matrix in eq 1 can be approximated to zero, which is called Tamm-Dancoff approximation (TDA).

**Figure 2:**
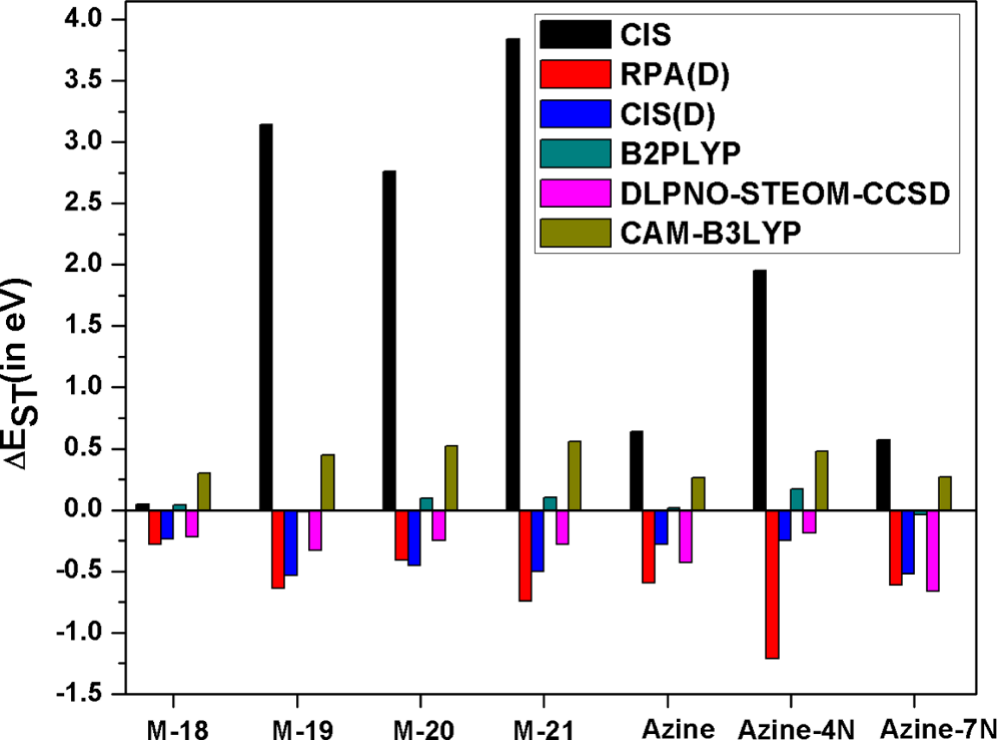
Single-triplet gaps (Δ*E*_ST_) for all seven chromophores were computed with different electronic structure methods using def2-TZVP basis sets.

As shown in Figure 2, both CIS and LR-TDDFT methods that only consider the single excitation space predict positive singlet–triplet gaps for all seven molecules. Interestingly, all types of exchange–correlation functionals demonstrate positive singlet–triplet gaps (Δ*E*_ST_ > 0) with the conventional LR-TDDFT method (see Table S1 in Supporting Information). In Figure 2, we have shown CAM-B3LYP as a representative example of the performance of exchange–correlation functionals within the LR-TDDFT regime.

Within a single reference framework, previous studies have shown that, unlike KS-DFT, correlated wave function methods have successfully predicted the inversion of singlet–triplet gaps for these types of molecules. In Figure 2, we have shown singlet–triplet gaps of all seven molecules with four wave function-based methods CIS, RPA(D), CIS(D), and DLPNO–STEOM–CCSD methods. RPA(D), CIS(D), and DLPNO–STEOM–CCSD successfully predicted inverted singlet–triplet gaps for all seven molecules (Figure 2).^30^ The difference between CIS and CIS(D) results clearly shows that correlation due to the double excitations needs to be accounted for during the calculation to get inverted singlet–triplet gaps. The success of the higher-level correlated methods like DLPNO–STEOM–CCSD in predicting this property can be attributed to correlation originating from higher-order excitations. The discrepancy between the performance of CIS and CIS(D) methods indicates that the failure of conventional LR-TDDFT to take double excitations or even the correlation resulting from double excitations into account can be a major reason for its failure.

**Figure 3:**
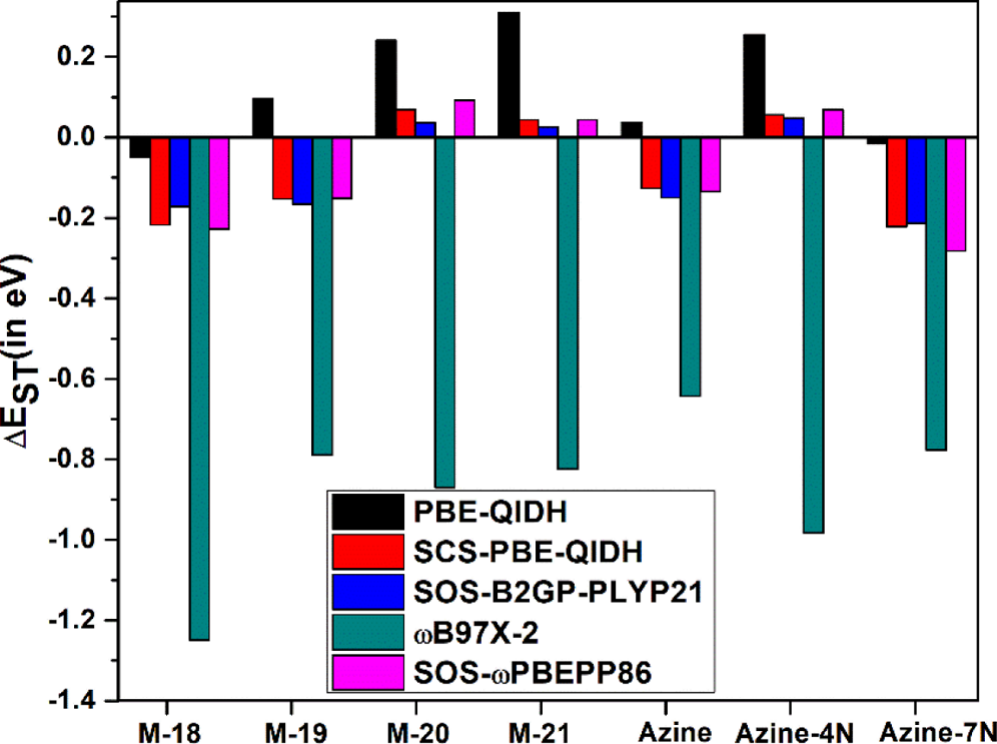
Single–triplet gaps (Δ*E*_ST_) for all seven chromophores were computed with different double-hybrid functionals using def2-TZVP basis sets.

In the same philosophy of CIS(D) or RPA(D), the LR-TDDFT(D) method can introduce the correlation originating from double excitation but such a method is only consistent with the concept of double-hybrid functionals, as introduced by Neese and Grimme.^51^ As shown in Figure 2, one of the most widely used double-hybrid functional, B2PLYP when applied with LR-TDDFT(D) produced mixed results as it has predicted negative Δ*E*_ST_ gaps only for **azine-7N** and **M-19**, isoenergetic Δ*E*_ST_ gap for azine but predicted positive Δ*E*_ST_ gaps for the remaining four molecules. Apart from different ground-state reference orbitals, RPA(D) and LR-TDDFT(D) used for double-hybrid functionals differ by the scaling factor for the exact exchange integral introduced by the functional form (eq 5) and also by the exchange–correlation kernel (*f̂*_xc_) (eqs 2 and 3). The nonlocal correlation part in B2PLYP (i.e., MP2 contribution) is scaled according to the functional form (eqs 4 and 5). RPA(D) predicts negative singlet–triplet gaps for all of the test cases, while B2PLYP only predicts negative singlet–triplet gaps for two systems. Clearly, it indicates that the origin of this difference lies in the functional form of B2PLYP. However, these results are truly encouraging as many previous studies concluded that it is not possible for KS-DFT exchange–correlation functionals to predict negative singlet–triplet gaps for such systems, but our study has shown otherwise. These results have shown that it is possible to get inverted Δ*E*_ST_ if proper functional form is chosen. With that in mind, we have tested a series of double-hybrid functionals for the seven molecules in our test set (Table S4). ωB2PLYP functional previously used by Pollice et al. provides negative Δ*E*_ST_ value only for one of the seven molecules (Table S4). Δ*E*_ST_ values for seven molecules obtained using some of the functionals have been reported in Figure 3. Like B2PLYP, PBE-QIDH^53^ provided mixed success for Δ*E*_ST_ values. ωB97X-2^54^ is the only functional that has predicted negative Δ*E*_ST_ gap for all seven molecules, but it has overestimated the Δ*E*_ST_ values for all of them. SOS-B2GP-PLYP21,^55−57^ SOS-ωPBEPP86,^57^ and SCS-PBE-QIDH^57^ functionals that were optimized specifically for excited states by Casanova-Páez and Goerigk provided either close to zero or negative Δ*E*_ST_ gap for all seven molecules. Looking at the results presented in Figure 3 and Table S5, it is safe to say that functionals like B2PLYP and ωB2PLYP only show mixed success for inverted singlet–triplet gaps because of the low value of *a*_c_ parameter. From our analysis, it is clear that for successful prediction of inverted singlet–triplet gap, a double-hybrid functional should have *a*_c_ ≥ 0.45, while they should also have *a*_x_ ≥ 0.50.

### 3.2 Multireference Wave Function Methods

All of the single reference methods studied here, despite their success, are only applicable for excited states that are dominated by single excitations. However, considering the important role that the doubles correlation plays in the singlet–triplet energetics of this set of molecules, the ideal method should treat both single and double excited states with equal accuracy. Multiconfiguration self-consistent field (MCSCF) methods like—CASSCF and RASSCF—are such theories that can treat both single and doubly excited states with similar accuracies. It is possible to calculate the percentage of doubly excited configurations in the first singlet excited state from MCSCF calculations. However, multireference character of the ground state has to be taken into account while estimating double excitation character of the S1 state.^32,58^ The HF configuration contributes ∼80% to the ground-state RASSCF wave function for all seven molecules. The S_1_ excited state is predominately single reference in nature with singly excited configuration contributing ∼73–80% to the first excited state. However, in all seven molecules, doubly excited configurations contribute ∼5% to the RASSCF wave function of the S_1_ excited states. Our analysis of the MCSCF wave function shows that even though doubly excited configurations have significant contribution to the S_1_ excited states of these molecules, the S_1_ excited state is still predominately single reference in nature.

MRPT2^59^ can treat both singly and doubly excited states with similar accuracy. To examine the performance of density functional theory for inverted singlet–triplet gaps when multireference wave function is used, we have studied the performance of MC-PDFT. Unlike the TDDFT(D) approach, in multireference wave function, doubly excited configurations are explicitly added in the excited-state wave function. Both MC-PDFT and MRPT2 methods are based on the wave functions obtained from the MCSCF calculations. While MRPT2 recovers dynamical correlation using PT2, MC-PDFT recovers dynamical correlation using a new type of exchange–correlation functionals called on-top pair-density functionals.^35^ Total MC-PDFT energy of Ψ_MCSCF_ state
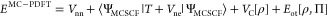
6where *V*_nn_ is the nuclear repulsion energy, ⟨Ψ_MCSCF_|*T* + *V*_ne_|Ψ_MCSCF_⟩ is the summation of kinetic and nuclear-electron attraction energies obtained from MCSCF calculations, *V*_C_[ρ] is the classical electron–electron repulsion energy, and *E*_ot_[ρ,Π] is the on-top pair-density energy.

In our calculations, we have used a variation of the MCSCF method, RASSCF method, and the corresponding PT2 method, RASPT2, for studying the excited states of the systems in the test set (Figure 1). RASPT2 predicted singlet–triplet gaps of all molecules to be either negative or close to zero, which is qualitatively similar to correlated single reference wave function methods like CIS(D) and STEOM-CCSD. Interestingly, the on-top density functionals only predicted negative singlet–triplet gaps for **M19** and **M21** (see Tables 1 and S3), even though MC-PDFT uses the same reference wave function as RASPT2. We examined contributions of individual component to singlet–triplet gaps of all seven molecules in Table 2. First two terms in the MC-PDFT energy expression is the same as MCSCF, and the classical electron–electron repulsion term is exact. In Table 2, we see that contributions of one electron energy and classical electron–electron repulsion energy to the singlet–triplet gaps are opposite in sign in all cases. Correlation energy contributes very little to the singlet–triplet energy gaps. However, exchange energy has a very significant contribution to the singlet–triplet energy gaps and possibly the reason behind the mixed success of MC-PDFT. This aspect will be discussed later in the context of eDFT results.

**Table 1:** Vertical S_0_–S_1_ and S_0_–T_1_ Excitation Energies (in eV) of the Studied Chromophores Based on Multireference Calculations

	**RASSCF**			**RASPT2**			**tPBE**			**DLPNO–STEOM–CCSD**		
	**S**_**1**_	**T**_**1**_	**Δ*E***_**ST**_	**S**_**1**_	**T**_**1**_	**Δ*E***_**ST**_	**S**_**1**_	**T**_**1**_	**ΔE**_**ST**_	**S**_**1**_	**T**_**1**_	**Δ*E***_**ST**_
**M18**	2.03	2.27	**–0.24**	1.98	1.92	**0.06**	2.29	2.12	**0.17**	1.83	2.05	**–0.22**
**M19**	2.51	2.61	**–0.10**	2.56	2.62	**–0.06**	2.91	2.96	**–0.05**	2.31	2.64	**–0.33**
**M20**	2.35	2.59	**–0.24**	2.52	2.58	**–0.06**	2.97	2.83	**0.14**	2.28	2.53	**–0.25**
**M21**	2.71	2.86	**–0.15**	2.76	2.92	**–0.16**	3.08	3.22	**–0.14**	2.47	2.75	**–0.28**
**azine**	0.65	0.93	**–0.28**	0.86	0.89	**–0.03**	1.37	1.2	**0.17**	0.61	1.04	**–0.43**
**azine-4N**	2.24	2.22	**0.02**	1.89	1.84	**0.05**	2.03	1.94	**0.09**	1.92	2.11	**–0.19**
**azine-7N**	2.56	2.95	**–0.39**	2.54	2.67	**–0.13**	2.8	2.69	**0.11**	2.35	3.01	**–0.66**

**Table 2:** Contributions of Different Components of MC-PDFT Energy to the Vertical Singlet–Triplet Gaps (in eV) of the Studied Chromophores Calculated with tPBE Functional

	one-electron energy	classical e–e repulsion energy	exchange energy	correlation energy	Δ*E*_ST_
**M-18**	0.59	–0.09	–0.35	0.02	0.17
**M-19**	–0.42	0.31	0.07	–0.01	0.05
**M-20**	–0.64	0.90	–0.13	0.01	0.13
**M-21**	–0.56	0.20	0.24	–0.02	–0.13
**azine**	–0.96	1.50	–0.38	0.01	0.17
**azine-4N**	–0.89	0.68	0.31	–0.01	0.09
**azine-7N**	–1.10	0.95	0.27	0.00	0.11

### 3.3 Excited-State DFT

Within the framework of KS-DFT, here, we have further tested the singlet–triplet gaps using a variational excited-state method called eDFT. In this approach, we have a variationally optimized ground (S_0_) and first triplet excited state (T_1_) using regular SCF procedure in the ground-state KS-DFT framework. For the S_1_ state, we have modified the SCF procedure at every density matrix update step by keeping the occupation number of beta HOMO and beta LUMO 0 and 1, respectively. We have obtained the energy of the S_1_ state for all seven molecules in our test set based on this approach.

**Table 3:** Vertical S_0_–S_1_ and S_0_–T_1_ Excitation Energies (in eV) of the Studied Chromophores Based on eDFT Calculations

	PBE			B3LYP			PBE0			HF			DLPNO–STEOM–CCSD		
	S_1_	T_1_	Δ*E*_ST_	S_1_	T_1_	Δ*E*_ST_	S_1_	T_1_	Δ*E*_ST_	S_1_	T_1_	Δ*E*_ST_	S_1_	T_1_	Δ*E*_ST_
**M18**	2.05	2.03	**0.02**	2.02	2.06	**–0.04**	1.99	2.22	**–0.23**	0.53	1.51	**–0.97**	1.83	2.05	**–0.22**
**M19**	2.57	2.53	**0.04**	2.59	2.60	**–0.01**	2.58	2.62	**–0.03**	3.26	2.23	**1.03**	2.31	2.64	**–0.33**
**M20**	2.54	2.53	**0.01**	2.56	2.53	**0.03**	2.55	2.53	**0.02**	3.02	2.09	**0.93**	2.28	2.53	**–0.25**
**M21**	2.76	2.75	**0.01**	2.79	2.88	**–0.08**	2.79	2.93	**–0.13**	1.48	2.15	**–0.67**	2.47	2.75	**–0.28**
**azine**	1.06	1.07	**–0.01**	0.96	1.07	**–0.11**	0.89	1.06	**–0.17**	0.78	1.74	**–0.95**	0.61	1.04	**–0.43**
**azine-4N**	1.91	1.89	**0.03**	1.94	2.00	**–0.07**	1.92	2.04	**–0.11**	1.32	2.52	**–1.20**	1.92	2.11	**–0.19**
**azine-7N**	2.52	2.54	**–0.02**	2.59	2.75	**–0.15**	2.59	2.82	**–0.22**	1.43	3.75	**–2.32**	2.35	3.01	**–0.66**

We have calculated singlet–triplet gaps for all seven molecules in our test set using B3LYP, PBE,^60^ and PBE0^61^ functional within eDFT method (Table 3). We have also computed the singlet–triplet gaps using the eDFT approach for HF theory. Interestingly, all three density functionals predict near zero or negative singlet–triplet gaps, but HF theory overestimates the singlet–triplet gaps. Computed singlet–triplet gaps increase with the amount of HF exchange in the exchange–correlation functional. Among all three functionals tested here, PBE0 predicts singlet–triplet gap closest to the DLPNO–STEOM–CCSD results.

These results are particularly encouraging because LR-TDDFT fails to get correct singlet–triplet gaps for these seven molecules using the same functionals. This points to the lack of orbital optimization at the excited-state as the origin of the failure of LR-TDDFT for inverted singlet–triplet gaps.

As tPBE pair-density functional was developed from PBE KS-DFT exchange–correlation functional, we explore the results obtained using PBE in more details. In Table 4, we have reported the decomposition of singlet–triplet energy gaps for different components of KS-DFT energy equation. Interestingly, exchange energy contributions to the singlet–triplet gaps computed from eDFT are always negative. However, in the case of tPBE, the contribution from the exchange energy does not always go in the same direction. We have seen that in the case of tPBE, for M-19, M-21, azine-4N, and azine-7N, the exchange energy contributions are positive, whereas exchange energy turns negative for M-18, M-20, and azine (Table 2). It is possible that new pair-density functionals need to be developed to get accurate results for these systems.^62,63^ It is also important to note that MC-PDFT is a post-SCF process like LR-TDDFT. Hence, the lack of orbital optimization may also be a reason for the failure of MC-PDFT for inverted singlet–triplet gaps.

**Table 4:** Contributions of Different Components of KS-DFT Energy to the Vertical Singlet-Triplet Gaps (in eV) of the Studied Chromophores Calculated with PBE Functional Using the eDFT Method

	one-electron energy	classical e–e repulsion energy	exchange energy	correlation energy	Δ*E*_ST_
**M-18**	–0.72	0.83	–0.09	0.01	0.02
**M-19**	–1.38	1.42	–0.01	0.01	0.04
**M-20**	–0.77	0.89	–0.12	0.01	0.01
**M-21**	–1.18	1.28	–0.10	0.01	0.01
**azine**	–1.07	1.23	–0.19	0.02	–0.01
**azine-4N**	–0.51	0.62	–0.09	0.01	0.03
**azine-7N**	–1.15	1.35	–0.25	0.02	–0.02

## 4 Conclusions

In this article, we have tested different density functional and combined density functional and wave function theories to determine the origin of the failure of density functionals for inverted singlet–triplet gaps. We have found that the LR-TDDFT(D) method with proper choice of double-hybrid density functionals can obtain singlet–triplet inversion. These results indicate that the inclusion of correlation resulting from double excitations is essential to get inverted singlet–triplet gaps. Our MCSCF calculations, however, have shown that even though first excited states of all seven molecules studied here have non-zero contributions of doubly excited configurations, these excited states are dominated mainly by a singly excited configuration. These results show that the inclusion of doubly excited configurations to the excited state wave function is not always important for accurate energetics of the singlet and triplet states. Although RASSCF and RASPT2 provided correct energetics for inverted singlet–triplet gaps, MC-PDFT with the same RASSCF wave function provided mixed success for the same set of molecules.

To see the effect of orbital optimization on the excited-state energetics, we have calculated singlet–triplet energies of our test set using the eDFT method. We found that eDFT with conventional local and hybrid functionals produced correct energetics for the studied molecules. These results indicate toward the importance of orbital optimization for obtaining correct singlet–triplet gaps in these molecules, which is missing in the LR-TDDFT.

Finally, we conclude that it is possible to obtain inverted singlet–triplet gaps using the DFT framework either by using the LR-TDDFT(D) method with proper choice of double-hybrid functionals or by using the eDFT method as a final nail in the coffin about the computation of inverted singlet–triplet gaps.

## Supporting Information Available

Excitation energies and singlet–triplet gaps calculated with different KS-DFT and pair-density functionals, orbitals included in the RASSCF active space, and their occupation numbers for S_0_, S_1_, and T_1_ wave functions and coordinates of the optimized structures (PDF)

## References

[ref1] Nakano M. Excitation Energies and Properties of Open-Shell Singlet Molecules Applications to a New Class of Molecules for Nonlinear Optics and Singlet Fission; Maroulis G., Ed.; Springer: Heidelberg, 2014.

[ref2] Feringa B. L.; van Delden R. A.; Koumura N.; Geertsema E. M. Chiroptical Molecular Switches. Chem. Rev. 2000, 100, 1789–1816. 10.1021/cr9900228. 11777421

[ref3] Sato K.; Shizu K.; Yoshimura K.; Kawada A.; Miyazaki H.; Adachi C. Organic luminescent molecule with energetically equivalent singlet and triplet excited states for organic light-emitting diodes. Phys. Rev. Lett. 2013, 110, 247401 10.1103/physrevlett.110.247401. 25165959

[ref4] Nobuyasu R. S.; Ren Z.; Griffiths G. C.; Batsanov A. S.; Data P.; Yan S.; Monkman A. P.; Bryce M. R.; Dias F. B. Rational Design of TADF Polymers Using a Donor-Acceptor Monomer with Enhanced TADF Efficiency Induced by the Energy Alignment of Charge Transfer and Local Triplet Excited States. Adv. Opt. Mater. 2016, 4, 597–607. 10.1002/adom.201500689.

[ref5] Dias F. B.; Santos J.; Graves D. R.; Data P.; Nobuyasu R. S.; Fox M. A.; Batsanov A. S.; Palmeira T.; Berberan-Santos M. N.; Bryce M. R.; et al. The role of local triplet excited states and D-A relative orientation in thermally activated delayed fluorescence: Photophysics and devices. Adv. Sci. 2016, 3, 1600080 10.1002/advs.201600080. PMC5157178 27981000

[ref6] Samanta P. K.; Kim D.; Coropceanu V.; Brédas J.-L. Up-Conversion Intersystem Crossing Rates in Organic Emitters for Thermally Activated Delayed Fluorescence: Impact of the Nature of Singlet vs Triplet Excited States. J. Am. Chem. Soc. 2017, 139, 4042–4051. 10.1021/jacs.6b12124. 28244314

[ref7] Endo A.; Sato K.; Yoshimura K.; Kai T.; Kawada A.; Miyazaki H.; Adachi C. Efficient up-conversion of triplet excitons into a singlet state and its application for organic light emitting diodes. Appl. Phys. Lett. 2011, 98, 083302 10.1063/1.3558906.

[ref8] Uoyama H.; Goushi K.; Shizu K.; Nomura H.; Adachi C. Highly efficient organic light-emitting diodes from delayed fluorescence. Nature 2012, 492, 234–238. 10.1038/nature11687. 23235877

[ref9] Liu Y.; Li C.; Ren Z.; Yan S.; Bryce M. R. All-organic thermally activated delayed fluorescence materials for organic light-emitting diodes. Nat. Rev. Mater. 2018, 3, 18020 10.1038/natrevmats.2018.20.

[ref10] Yang Z.; Mao Z.; Xie Z.; Zhang Y.; Liu S.; Zhao J.; Xu J.; Chi Z.; Aldred M. P. Recent advances in organic thermally activated delayed fluorescence materials. Chem. Soc. Rev. 2017, 46, 915–1016. 10.1039/c6cs00368k. 28117864

[ref11] Chen X.-K.; Kim D.; Brédas J.-L. Thermally activated delayed fluorescence (TADF) path toward efficient electroluminescence in purely organic materials: molecular level insight. Acc. Chem. Res. 2018, 51, 2215–2224. 10.1021/acs.accounts.8b00174. 30141908

[ref12] de Silva P.; Kim C. A.; Zhu T.; Van Voorhis T. Extracting design principles for efficient thermally activated delayed fluorescence (TADF) from a simple four-state model. Chem. Mater. 2019, 31, 6995–7006. 10.1021/acs.chemmater.9b01601.

[ref13] Ehrmaier J.; Rabe E. J.; Pristash S. R.; Corp K. L.; Schlenker C. W.; Sobolewski A. L.; Domcke W. Singlet-Triplet Inversion in Heptazine and in Polymeric Carbon Nitrides. J. Phys. Chem. A 2019, 123, 8099–8108. 10.1021/acs.jpca.9b06215. 31466450

[ref14] de Silva P. Inverted Singlet–Triplet Gaps and Their Relevance to Thermally Activated Delayed Fluorescence. J. Phys. Chem. Lett. 2019, 10, 5674–5679. 10.1021/acs.jpclett.9b02333. 31483656

[ref15] Pios S.; Huang X.; Sobolewski A. L.; Domcke W. Triangular boron carbon nitrides: An unexplored family of chromophores with unique properties for photocatalysis and ptoelectronics. Phys. Chem. Chem. Phys. 2021, 23, 12968–12975. 10.1039/d1cp02026a. 34059871

[ref16] Sobolewski A. L.; Domcke W. Are Heptazine-Based Organic Light-Emitting Diode Chromophores Thermally Activated Delayed Fluorescence or Inverted Singlet-Triplet Systems?. J. Phys. Chem. Lett. 2021, 12, 6852–6860. 10.1021/acs.jpclett.1c01926. 34279950

[ref17] Pollice R.; Friederich P.; Lavigne C.; Gomes G. d. P.; Aspuru-Guzik A. Organic Molecules with Inverted Gaps between First Excited Singlet and Triplet States and Appreciable Fluorescence Rates. Matter 2021, 4, 1654–1682. 10.1016/j.matt.2021.02.017.

[ref18] Hund F. Zur deutung verwickelter spektren, insbesondere der elemente scandium bis nickel. Physik 1925, 33, 345–371. 10.1007/bf01328319.

[ref19] Leupin W.; Wirz J. Low-Lying Electronically Excited States of Cycl[3.3.3]azine, a Bridged 12π-Perimeter. J. Am. Chem. Soc. 1980, 102, 6068–6075. 10.1021/ja00539a016.

[ref20] Leupin W.; Magde D.; Persy G.; Wirz J. 1, 4, 7-Triazacycl [3.3. 3] azine: basicity, photoelectron spectrum, photophysical properties. J. Am. Chem. Soc. 1986, 108, 17–22. 10.1021/ja00261a004.

[ref21] Rhee Y. M.; Head-Gordon M. Scaled Second-Order Perturbation Corrections to Configuration Interaction Singles: Efficient and Reliable Excitation Energy Methods. J. Phys. Chem. A 2007, 111, 5314–5326. 10.1021/jp068409j. 17521172

[ref22] Runge E.; Gross E. K. U. Density-Functional Theory for Time Dependent Systems. Phys. Rev. Lett. 1984, 52, 997–1000. 10.1103/physrevlett.52.997.

[ref23] Dinkelbach F.; Bracker M.; Kleinschmidt M.; Marian C. M. Large Inverted Singlet–Triplet Energy Gaps Are Not Always Favorable for Triplet Harvesting: Vibronic Coupling Drives the (Reverse) Intersystem Crossing in Heptazine Derivatives. J. Phys. Chem. A 2021, 125, 10044–10051. 10.1021/acs.jpca.1c09150. 34756038

[ref24] Hellweg A.; Grün S. A.; Hättig C. Benchmarking the ¨ performance of spin-component scaled CC2 in ground and electronically excited states. Phys. Chem. Chem. Phys. 2008, 10, 4119–4127. 10.1039/b803727b. 18612515

[ref25] Wormit M.; Rehn D. R.; Harbach P. H. P.; Wenzel J.; Krauter C. M.; Epifanovsky E.; Dreuw A. Investigating excited electronic states using the algebraic diagrammatic construction (ADC) approach of the polarisation propagator. Mol. Phys. 2014, 112, 774–784. 10.1080/00268976.2013.859313.

[ref26] Angeli C.; Cimiraglia R.; Evangelisti S.; Leininger T.; Malrieu J.-P. Introduction of N-electron valence states for multireference perturbation theory. J. Chem. Phys. 2001, 114, 10252–10264. 10.1063/1.1361246.

[ref27] Ricci G.; San-Fabián E.; Olivier Y.; Sancho-García J. C. Singlet-Triplet Excited-State Inversion in Heptazine and Related Molecules: Assessment of TD-DFT and ab initio Methods. Chem. Phys. Chem. 2021, 22, 553–560. 10.1002/cphc.202000926. 33325598

[ref28] Sanz-Rodrigo J.; Ricci G.; Olivier Y.; Sancho-García J. C. Negative Singlet–Triplet Excitation Energy Gap in Triangle-Shaped Molecular Emitters for Efficient Triplet Harvesting. J. Phys. Chem. A 2021, 125, 513–522. 10.1021/acs.jpca.0c08029. 33401898

[ref29] Nooijen M.; Bartlett R. J. Similarity transformed equation-of-motion coupled-cluster theory: Details, examples, and comparisons. J. Chem. Phys. 1997, 107, 6812–6830. 10.1063/1.474922.

[ref30] Bhattacharyya K. Can TDDFT Render the Electronic Excited States Ordering of Azine Derivative? A Closer Investigation with DLPNO-STEOM-CCSD. Chem. Phys. Lett. 2021, 779, 138827 10.1016/j.cplett.2021.138827.

[ref31] Miyajima D.; Aizawa N.; Pu Y.-J.; Nihonyanagi A.; Ibuka R.; Inuzuka H.; Dhara B.; Koyama Y.; Araoka F. Delayed Fluorescence from Inverted Singlet and Triplet Excited States for Efficient Organic Light-Emitting Diodes. Phys. Sci. 2021, 10.21203/rs.3.rs-478258/v1. PMC9477729 36104553

[ref32] Elliott P.; Goldson S.; Canahui C.; Maitra N. T. Perspectives on double-excitations in TDDFT. Chem. Phys. 2011, 391, 110–119. 10.1016/j.chemphys.2011.03.020.

[ref33] Head-Gordon M.; Rico R. J.; Oumi M.; Lee T. J. A doubles correction to electronic excited states from configuration interaction in the space of single substitutions. Chem. Phys. Lett. 1994, 219, 21–29. 10.1016/0009-2614(94)00070-0.

[ref34] Ghosh S.; Verma P.; Cramer C. J.; Gagliardi L.; Truhlar D. G. Combining Wave Function Methods with Density Functional Theory for Excited States. Chem. Rev. 2018, 118, 7249–7292. 10.1021/acs.chemrev.8b00193. 30044618

[ref35] Li Manni G.; Carlson R. K.; Luo S.; Ma D.; Olsen J.; Truhlar D. G.; Gagliardi L. Multi-Configuration Pair-Density Functional Theory. J. Chem. Theory Comput. 2014, 10, 3669–3680. 10.1021/ct500483t. 26588512

[ref36] Hait D.; Head-Gordon M. Orbital optimized density functional theory for electronic excited states. J. Phys. Chem. Lett. 2021, 12, 4517–4529. 10.1021/acs.jpclett.1c00744. 33961437

[ref37] Ziegler T.; Rauk A.; Baerends E. J. On the calculation of multiplet energies by the hartree-fock-slater method. Theor. Chim. Acta 1977, 43, 261–271. 10.1007/bf00551551.

[ref38] Cheng C.-L.; Wu Q.; Van Voorhis T. Rydberg energies using excited state density functional theory. J. Chem. Phys. 2008, 129, 124112 10.1063/1.2977989. 19045011

[ref39] Artacho E.; Rohlfing M.; Côté M.; Haynes P. D.; Needs R. J.; Molteni C. Structural Relaxations in Electronically Excited Poly(para-phenylene). Phys. Rev. Lett. 2004, 93, 116401 10.1103/physrevlett.93.116401. 15447360

[ref40] Ceresoli D.; Tosatti E.; Scandolo S.; Santoro G.; Serra S. Trapping of excitons at chemical defects in polyethylene. J. Chem. Phys. 2004, 121, 6478 10.1063/1.1783876. 15446948

[ref41] Pankratov O.; Scheffler M. Localized Excitons and Breaking of Chemical Bonds at III-V (110) Surfaces. Phys. Rev. Lett. 1995, 75, 701 10.1103/physrevlett.75.701. 10060092

[ref42] Kowalczyk T.; Yost S. R.; Voorhis T. V. Assessment of the ΔSCF Density Functional Theory Approach for Electronic Excitations in Organic Dyes. J. Chem. Phys. 2011, 134, 054128 10.1063/1.3530801. 21303113

[ref43] Neese F. Software Update: The Orca Program System, version 4.0; WIREs Comput. Mol. Sci., 2018.

[ref44] Olsen J.; Roos B. O.; Jo/rgensen P.; Jensen H. J. r. A. Determinant Based Configuration Interaction Algorithms for Complete and Restricted Configuration Interaction Spaces. J. Chem. Phys. 1988, 89, 2185–2192. 10.1063/1.455063.

[ref45] Malmqvist P. A.; Pierloot K.; Shahi A. R. M.; Cramer C. J.; Gagliardi L. The Restricted Active Space Followed by Second-Order Perturbation Theory Method: Theory and Application to the Study of CuO2 and Cu2O2 Systems. J. Chem. Phys. 2008, 128, 204109 10.1063/1.2920188. 18513012

[ref46] Fdez Galván I.; Vacher M.; Alavi A.; Angeli C.; Aquilante F.; Autschbach J.; Bao J. J.; Bokarev S. I.; Bogdanov N. A.; Carlson R. K.; Chibotaru L. F.; Creutzberg J.; Dattani N.; Delcey M. G.; Dong S. S.; Dreuw A.; Freitag L.; Frutos L. M.; Gagliardi L.; Gendron F.; Giussani A.; González L.; Grell G.; Guo M.; Hoyer C. E.; Johansson M.; Keller S.; Knecht S.; Kovačević G.; Källman E.; Li Manni G.; Lundberg M.; Ma Y.; Mai S.; Malhado J. P.; Malmqvist P. Å.; Marquetand P.; Mewes S. A.; Norell J.; Olivucci M.; Oppel M.; Phung Q. M.; Pierloot K.; Plasser F.; Reiher M.; Sand A. M.; Schapiro I.; Sharma P.; Stein C. J.; Sørensen L. K.; Truhlar D. G.; Ugandi M.; Ungur L.; Valentini A.; Vancoillie S.; Veryazov V.; Weser O.; Wesołowski T. A.; Widmark P.-O.; Wouters S.; Zech A.; Zobel J. P.; Lindh R. OpenMolcas: From Source Code to Insight. J. Chem. Theory Comput. 2019, 15, 5925–5964. 10.1021/acs.jctc.9b00532. 31509407

[ref47] Forsberg N.; Malmqvist P.-Å. Multiconfiguration perturbation theory with imaginary level shift. Chem. Phys. Lett. 1997, 274, 196–204. 10.1016/s0009-2614(97)00669-6.

[ref48] Weigend F.; Ahlrichs R. Balanced basis sets of split valence, triple zeta valence and quadruple zeta valence quality for H to Rn: Design and assessment of accuracy. Phys. Chem. Chem. Phys. 2005, 7, 3297–3305. 10.1039/b508541a. 16240044

[ref49] Becke A. D. Density-functional thermochemistry. III. The role of exact exchange. J. Chem. Phys. 1993, 98, 5648–5652. 10.1063/1.464913.

[ref50] Aprà E.; Bylaska E. J.; de Jong W. A.; Govind N.; Kowalski ` K.; Straatsma T. P.; Valiev M.; van Dam H. J. J.; Alexeev Y.; Anchell J.; et al. NWChem: Past, present, and future. J. Chem. Phys. 2020, 152, 184102 10.1063/5.0004997. 32414274

[ref51] Grimme S.; Neese F. Double-Hybrid Density Functional Theory for Excited Electronic States of Molecules. J. Chem. Phys. 2007, 127, 154116 10.1063/1.2772854. 17949141

[ref52] Ottochian A.; Morgillo C.; Ciofini I.; Frisch M. J.; Scalmani G.; Adamo C. Double hybrids and time-dependent density functional theory:An implementation and benchmark on charge transfer excitedstates. J. Comput. Chem. 2020, 41, 1242–1251. 10.1002/jcc.26170. 32073175

[ref53] Brémond E.; Sancho-García J. C.; Pérez-Jiménez ÁJ.; Adamo C. Communication: double-hybrid functionals from adiabatic-connection: the QIDH model. J. Chem. Phys. 2014, 141, 031101 10.1063/1.4890314. 25053294

[ref54] Chai J.-D.; Head-Gordon M. Long-range corrected double-hybrid density functionals. J. Chem. Phys. 2009, 131, 174105 10.1063/1.3244209. 19894996

[ref55] Schwabe T.; Goerigk L. Time-dependent double-hybrid density functionals with spin-component and spin-opposite scaling. J. Chem. Theory Comput. 2017, 13, 4307–4323. 10.1021/acs.jctc.7b00386. 28763220

[ref56] Casanova-Páez M.; Goerigk L. Assessing the Tamm–Dancoff approximation, singlet–singlet, and singlet–triplet excitations with the latest long-range corrected double-hybrid density functionals. J. Chem. Phys. 2020, 153, 064106 10.1063/5.0018354. 35287444

[ref57] Casanova-Páez M.; Goerigk L. Time-Dependent Long-Range-Corrected Double-Hybrid Density Functionals with Spin-Component and Spin-Opposite Scaling: A Comprehensive Analysis of Singlet-Singlet and Singlet-Triplet Excitation Energies. J. Chem. Theory Comput. 2021, 17, 5165–5186. 10.1021/acs.jctc.1c00535. 34291643

[ref58] Loos P.-F.; Boggio-Pasqua M.; Scemama A.; Caffarel M.; Jacquemin D. Reference energies for double excitations. J. Chem. Theory Comput. 2019, 15, 1939–1956. 10.1021/acs.jctc.8b01205. 30689951

[ref59] Hirao K. Multireference Møller-Plesset method. Chem. Phys. Lett. 1992, 190, 374–380. 10.1016/0009-2614(92)85354-d.

[ref60] Perdew J. P.; Burke K.; Ernzerhof M. Generalized Gradient Approximation Made Simple. Phys. Rev. Lett. 1996, 77, 3865–3868. 10.1103/physrevlett.77.3865. 10062328

[ref61] Adamo C.; Barone V. Toward Reliable Density Functional Methods Without Adjustable Parameters: The PBE0 Model. J. Chem. Phys. 1999, 110, 6158–6170. 10.1063/1.478522.

[ref62] Sharma P.; Truhlar D. G.; Gagliardi L. Active space dependence in multiconfiguration pair-density functional theory. J. Chem. Theory Comput. 2018, 14, 660–669. 10.1021/acs.jctc.7b01052. 29301088

[ref63] Sharma P.; Bernales V.; Truhlar D. G.; Gagliardi L. Valence ππ* excitations in benzene studied by multiconfiguration pair-density functional theory. J. Phys. Chem. Lett. 2018, 10, 75–81. 10.1021/acs.jpclett.8b03277. 30540476

