# Supplementary material for: Origin of the Failure of Density Functional Theories in Predicting Inverted Singlet–Triplet Gaps
Source: J Phys Chem A. 2022 Feb 11;126(8):1378–85. doi: 10.1021/acs.jpca.1c10492 (PMC8900124; doi:10.1021/acs.jpca.1c10492)
Supplement: Supplementary file 1 — jp1c10492_si_001.pdf [file jp1c10492_si_001.pdf]

SUPPORTING INFORMATION

Origin of The Failure of Density Functional Theories in Predicting Inverted Singlet-Triplet Gaps

Soumen Ghosh*, Kalishankar Bhattacharyya

Max-Planck-Institut für Kohlenforschung, D45470 Mülheim an der Ruhr, Germany

Email: chemsghosh@gmail.com

Content:

Table S1. TDDFT excitation energies for all seven molecules.	**S2**
Table S2. Excitation energies calculated with different wave-function method for all seven molecules.	**S2**
Table S3. Excitation energies calculated with different on-top pair-density functionals for all seven molecules	**S2-S3**
Table S4. ΔE_{ST} values calculated with different double-hybrid functionals using LR-TDDFT for all seven molecules	**S3**
Table S5. ΔE_{ST} values calculated with different double-hybrid functionals using TDA-TDDFT for all seven molecules	**S4**
Pictures and occupation numbers of orbitals involved in the active space of RASSCF calculations for all seven molecules	**S3-S10**
XYZ structures	**S11-S17**

Table S1. Vertical S_0-S_1, and S_0-T_1 excitation energies (in eV) of the studied chromophores based on DFT functional. Def2-TZVP basis are employed for all calculations.

Chromophores	PBE		B3LYP		CAM-B3LYP		wB97X-V		M06-2X		B2PLYP	
	S_1	T_1	S_1	T_1	S_1	T_1	S_1	T_1	S_1	T_1	S_1	T_1
M18	2.13	1.93	2.24	2.00	2.32	2.02	2.41	2.12	2.38	2.13	2.17	1.76
M19	2.67	2.44	2.84	2.53	3.00	2.55	3.13	2.71	3.04	2.72	2.73	2.42
M20	2.64	2.41	2.80	2.46	2.96	2.44	3.08	2.56	3.00	2.59	2.70	2.30
M21	2.85	2.62	3.05	2.68	3.23	2.67	3.36	2.90	3.27	2.93	2.93	2.40
Azine	1.17	1.00	1.24	1.05	1.25	0.99	1.28	1.03	1.24	1.03	1.11	1.03
Azine-4N	2.07	1.80	2.29	1.97	2.44	1.96	2.53	2.05	2.27	1.96	2.19	1.91
Azine-7N	2.70	2.52	2.96	2.74	3.19	2.92	3.29	3.04	3.19	2.97	2.75	3.08

Table S2. Vertical S_0-S_1, and S_0-T_1 excitation energies (in eV) of the studied chromophores based on wave-function method. Def2-TZVP basis are employed for all calculations.

Chromophores	CIS		CIS(D)		B2PLYP		DLPNO-STEOM-CCSD	
	S_1	T_1	S_1	T_1	S_1	T_1	S_1	T_1
M18	2.70	2.65	2.09	2.99	2.17	2.13	1.83	2.05
M19	3.66	0.52	2.55	3.19	2.74	2.75	2.31	2.64
M20	3.59	0.83	2.60	3.02	2.71	2.61	2.28	2.53
M21	3.98	0.14	2.81	3.54	2.93	2.83	2.47	2.75
Azine	1.64	1.00	0.91	1.12	1.12	1.10	0.61	1.04
Azine-4N	3.20	1.25	2.14	3.35	2.19	2.02	1.92	2.11

Azine-7N	4.35	3.78	2.49	3.10	2.75	2.79	2.35	3.01

Table S3. Vertical S_0-S_1, S_0-T_1 and ΔE_{ST} excitation energies (in eV) of the studied molecules calculated with different translated functionals. Def2-TZVP basis are employed for all calculations.

	**ftPBE**			**tBLYP**			**ftBLYP**		
	**S1**	**T1**	**ΔE_{ST}**	**S1**	**T1**	**ΔE_{ST}**	**S1**	**T1**	**ΔE_{ST}**
**M18**	2.24	2.12	0.12	2.25	2.09	0.16	2.22	2.11	0.11
**M19**	2.85	2.93	-0.08	2.89	2.93	-0.04	2.86	2.93	-0.07
**M20**	2.91	2.82	0.09	2.95	2.81	0.14	2.91	2.82	0.10
**M21**	3.03	3.19	-0.16	3.08	3.19	-0.12	3.04	3.19	-0.15
**Azine**	1.30	1.18	0.12	1.35	1.18	0.17	1.31	1.19	0.12
**Azine-4N**	2.00	1.94	0.06	2.04	1.94	0.10	2.01	1.95	0.07
**Azine-7N**	2.75	2.69	0.07	2.83	2.69	0.14	2.80	2.70	0.10

Table S4. ΔE_{ST} (in eV) between S1 and T1 excitation energies of the studied chromophores calculated using LR-TDDFT(D) method with various double-hybrid exchange-correlational functionals. Def2-TZVP basis are employed for all calculations.

Chromophores	B2PLYP	PBE-QIDH	SCS-PBE-QIDH	SOS-B2GP-PLYP	wB97X-2	wPBEPP86	SOS-wPBEPP86	wB88PP86	SCS-wB88PP86	ωB2PLYP
M18	0.04	-0.05	-0.22	-0.17	-1.25	-0.29	-0.23	-0.16	-0.14	0.89
M19	-0.01	0.10	-0.15	-0.17	-0.79	-0.17	-0.15	-0.06	-0.03	0.65
M20	0.10	0.24	0.07	0.04	-0.87	0.09	0.09	0.17	0.18	0.74
M21	0.11	0.31	0.04	0.03	-0.82	0.03	0.04	0.11	0.17	0.89
Azine	0.02	0.04	-0.13	-0.15	-0.64	-0.07	-0.14	-0.01	-0.07	0.20
Azine-4N	0.17	0.26	0.06	0.05	-0.98	0.06	0.07	0.16	0.15	0.58
Azine-7N	-0.04	-0.02	-0.22	-0.21	-0.78	-0.14	-0.28	-0.08	-0.18	-0.13

Table S5. ΔE_{ST} (in eV) between S1 and T1 excitation energies of the studied chromophores calculated using TDA-TDDFT(D) method with various double-hybrid exchange-correlational functionals. Def2-TZVP basis are employed for all calculations.

Chromophores	B2PLYP	PBE-QIDH	SCS-PBE-QIDH	SOS-B2GP-PLYP	ωB97X-2	ωPBEPP86	SOS-ωPBEPP86	wB88PP86	SCS-wB88PP86	ωB2PLYP
M18	0.08	0.10	-0.01	0.00	-0.33	0.04	-0.04	0.07	0.02	0.12
M19	0.01	0.05	-0.11	-0.07	-0.77	-0.07	-0.15	-0.01	-0.05	0.16
M20	0.06	0.15	0.00	0.02	-0.68	0.04	-0.02	0.10	0.07	0.29
M21	-0.02	0.10	-0.09	-0.06	-0.72	-0.07	-0.14	0.01	-0.03	0.27
Azine	0.02	0.04	-0.09	-0.08	-0.38	-0.02	-0.12	0.01	-0.05	0.05
Azine-4N	0.14	0.19	0.06	0.07	-0.43	0.11	0.03	0.16	0.11	0.23
Azine-7N	-0.04	-0.03	-0.21	-0.20	-0.66	-0.13	-0.27	-0.08	-0.17	-0.19

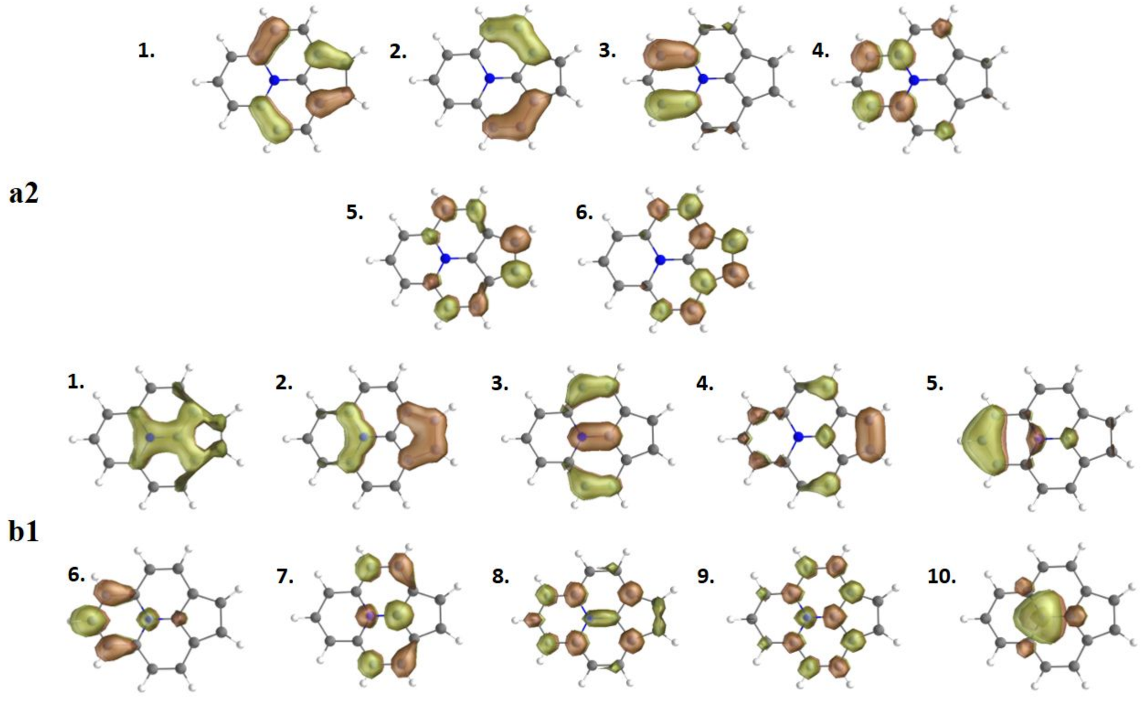

Figure S1. Pictures of orbital used in the active space for the RASSCF calculation of M-18 molecule.

Table S4. Orbital occupation numbers for S_0, S_1 and T_1 RASSCF wave function of M-18 molecule.

Symmetry	Orbital	Occupation (S_0)	Occupation (S_1)	Occupation (T_1)
b1	1	1.981288	1.98743	1.984839

	2	1.97422	1.966355	1.969486
	3	1.957772	1.965805	1.965391
	4	1.952375	1.926876	1.936794
	5	1.906657	1.890078	1.894756
	6	0.102393	0.993927	0.997234
	7	0.05967	0.083873	0.075394
	8	0.03725	0.037077	0.03521
	9	0.023153	0.028577	0.025736
	10	0.004625	0.005657	0.005757
a2	1	1.953705	1.922262	1.933773
	2	1.97052	1.964855	1.966984
	3	1.893477	1.016718	1.012632
	4	0.108531	0.116506	0.106172
	5	0.046822	0.059719	0.057671
	6	0.027544	0.034284	0.032172

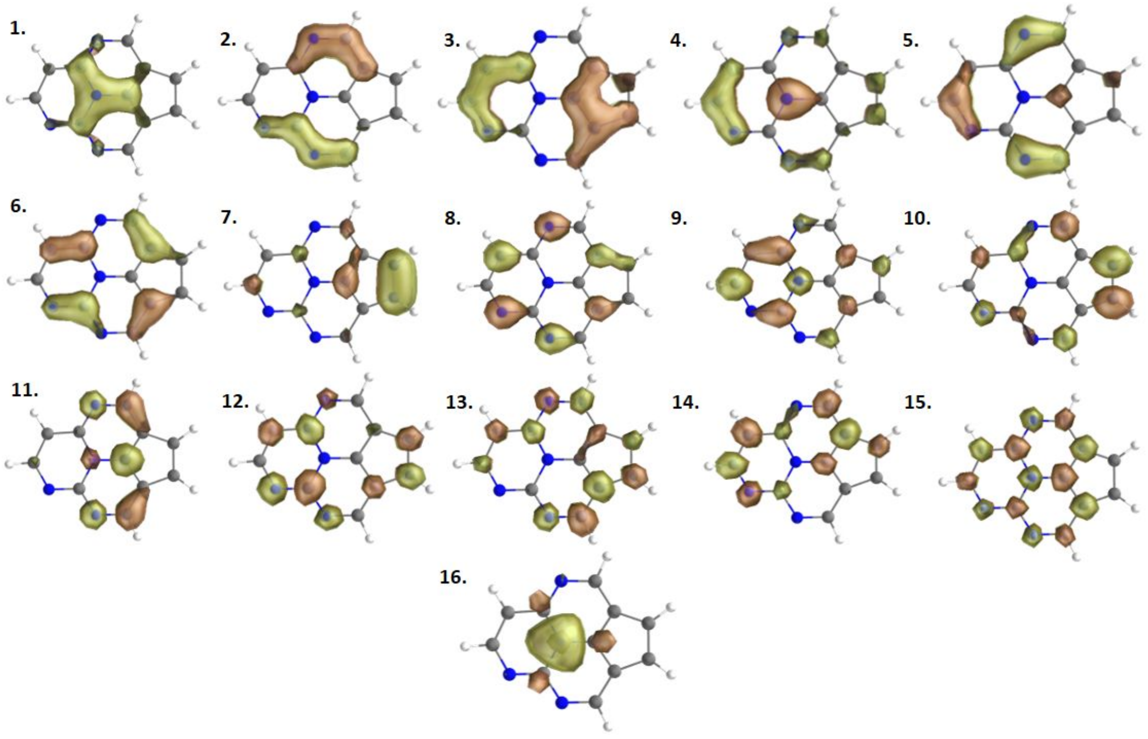

Figure S2. Pictures of orbital used in the active space for the RASSCF calculation of M-19 molecule.

Table S5. Orbital occupation numbers for S_0, S_1 and T_1 RASSCF wave function of M-19 molecule.

Symmetry	Orbital	Occupation (S_0)	Occupation (S_1)	Occupation (T_1)

a"	1	1.982792	1.987182	1.984318
	2	1.971171	1.961734	1.963222
	3	1.97352	1.966159	1.968975
	4	1.960692	1.963512	1.960018
	5	1.954132	1.924265	1.933636
	6	1.954412	1.920531	1.943371
	7	1.904784	1.886601	1.871734
	8	1.919623	1.050696	1.017049
	9	0.07721	0.959257	0.989545
	10	0.098529	0.120137	0.128482
	11	0.064027	0.086784	0.07577
	12	0.050999	0.063071	0.057452
	13	0.029968	0.03612	0.034078
	14	0.031484	0.038796	0.038463
	15	0.022472	0.029786	0.02846
	16	0.004185	0.005368	0.005428

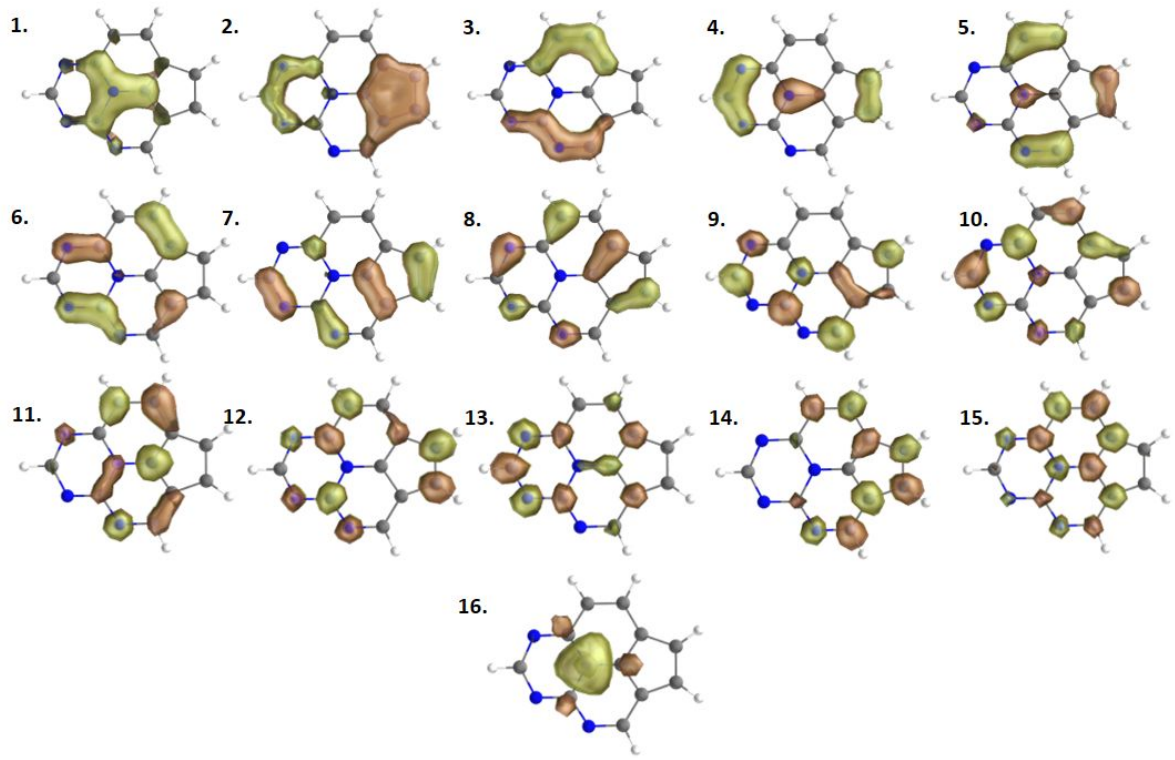

Figure S6. Pictures of orbital used in the active space for the RASSCF calculation of M-20 molecule.

Symmetry	Orbital	Occupation (S_0)	Occupation (S_1)	Occupation (T_1)
a"	1	1.983055	1.986753	1.984473

2	1.97433	1.967133	1.969247
3	1.971578	1.961614	1.964478
4	1.963366	1.95885	1.959676
5	1.950457	1.943039	1.929266
6	1.955087	1.919989	1.945819
7	1.91935	1.874258	1.889565
8	1.914458	1.032188	1.019581
9	0.071583	0.971852	0.989455
10	0.085895	0.126681	0.116298
11	0.07821	0.095697	0.07442
12	0.043976	0.048981	0.050623
13	0.035021	0.042372	0.041238
14	0.027241	0.034145	0.033204
15	0.022099	0.030927	0.027291
16	0.004295	0.005522	0.005367

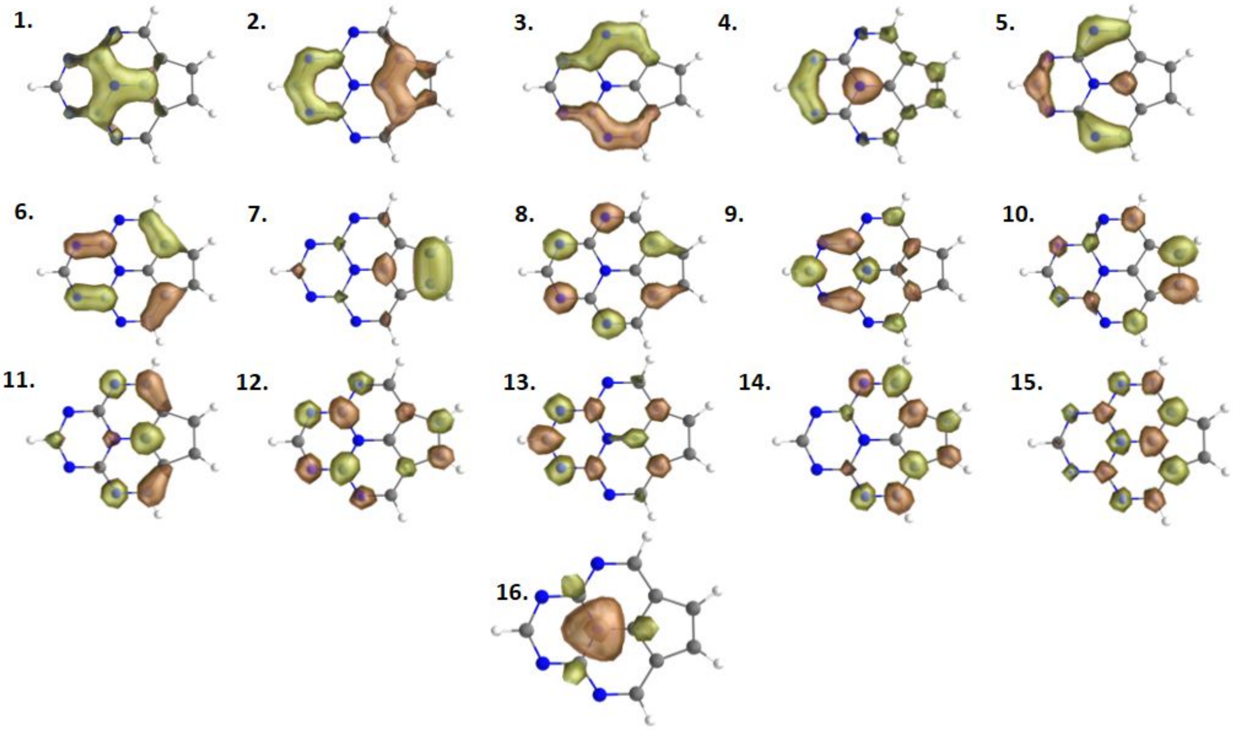

Figure S4. Pictures of orbital used in the active space for the RASSCF calculation of M-21 molecule.

Table S7. Orbital occupation numbers for S_0, S_1 and T_1 RASSCF wave function of M-21 molecule

Symmetry	Orbital	Occupation (S_0)	Occupation (S_1)	Occupation (T_1)

a”	1	1.983094	1.987536	1.984909
	2	1.973285	1.965871	1.967911
	3	1.971731	1.961203	1.962509
	4	1.961157	1.962785	1.960597
	5	1.95445	1.921956	1.93002
	6	1.95569	1.920433	1.945541
	7	1.904614	1.888195	1.877814
	8	1.925188	1.026329	1.016369
	9	0.070292	0.982802	0.990817
	10	0.097767	0.119499	0.123643
	11	0.062152	0.087097	0.07356
	12	0.050559	0.063849	0.054873
	13	0.033604	0.038821	0.040139
	14	0.029864	0.038206	0.036335
	15	0.022438	0.030151	0.029637
	16	0.004114	0.005267	0.005326

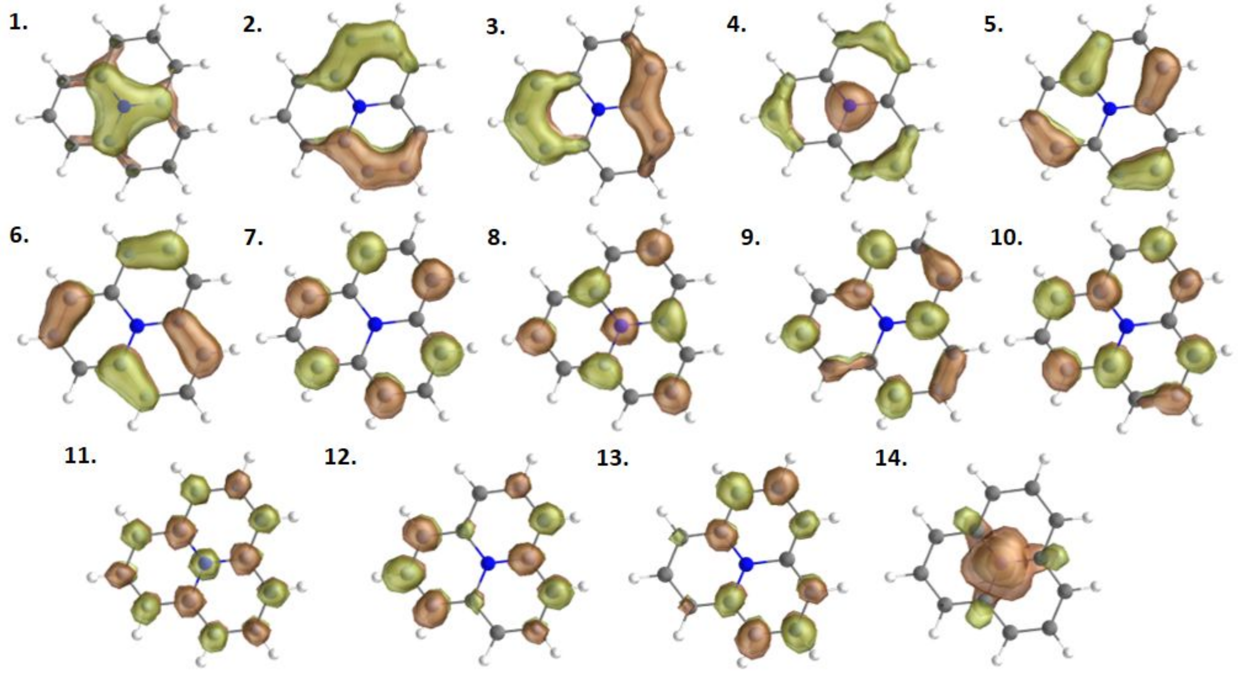

Figure S5. Pictures of orbital used in the active space for the RASSCF calculation of Azine molecule.

Table S8. Orbital occupation numbers for S_0, S_1 and T_1 RASSCF wave function of Azine molecule

Symmetry	Orbital	Occupation (S_0)	Occupation (S_1)	Occupation (T_1)

a1	1	1.983021	1.986973	1.985234
	2	1.972011	1.964789	1.967565
	3	1.972001	1.964775	1.966662
	4	1.958407	1.966388	1.967553
	5	1.949572	1.921577	1.930093
	6	1.949573	1.92158	1.930107
	7	1.901652	1.003631	0.996206
	8	0.082301	0.994913	0.99558
	9	0.070947	0.083541	0.080233
	10	0.070941	0.083529	0.080239
	11	0.021278	0.028642	0.025779
	12	0.031589	0.036636	0.034118
	13	0.031573	0.036614	0.03408
	14	0.005136	0.00641	0.006551

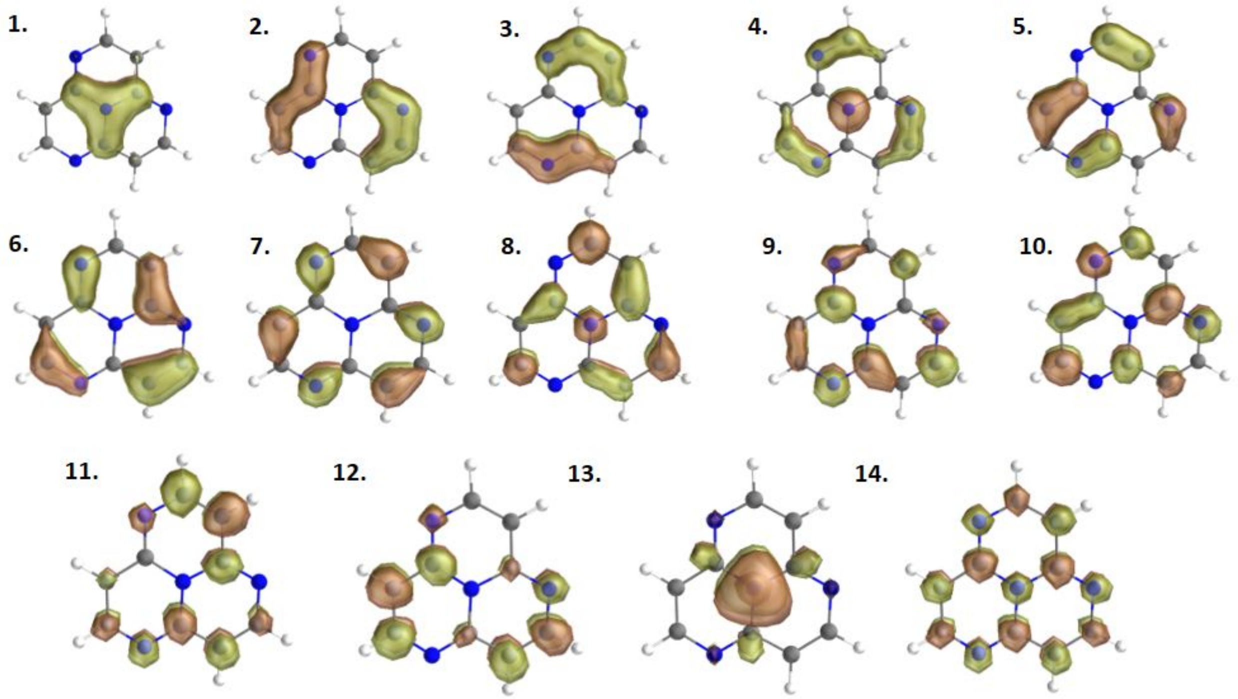

Figure S6. Pictures of orbital used in the active space for the RASSCF calculation of Azine-4N molecule.

Table S9. Orbital occupation numbers for S_0, S_1 and T_1 RASSCF wave function of Azine-4N molecule

Symmetry	Orbital	Occupation (S_0)	Occupation (S_1)	Occupation (T_1)
a”	1	1.983456	1.985855	1.985607
	2	1.970972	1.965821	1.964846

	3	1.970963	1.96581	1.964823
	4	1.961155	1.963925	1.963597
	5	1.951368	1.925651	1.924939
	6	1.951361	1.925635	1.924933
	7	1.922019	1.387132	1.023433
	8	0.064532	0.612646	0.972202
	9	0.064387	0.080293	0.084347
	10	0.064372	0.080286	0.084328
	11	0.033119	0.036314	0.036834
	12	0.033103	0.036294	0.03677
	13	0.004825	0.005614	0.005733
	14	0.024367	0.028723	0.027609

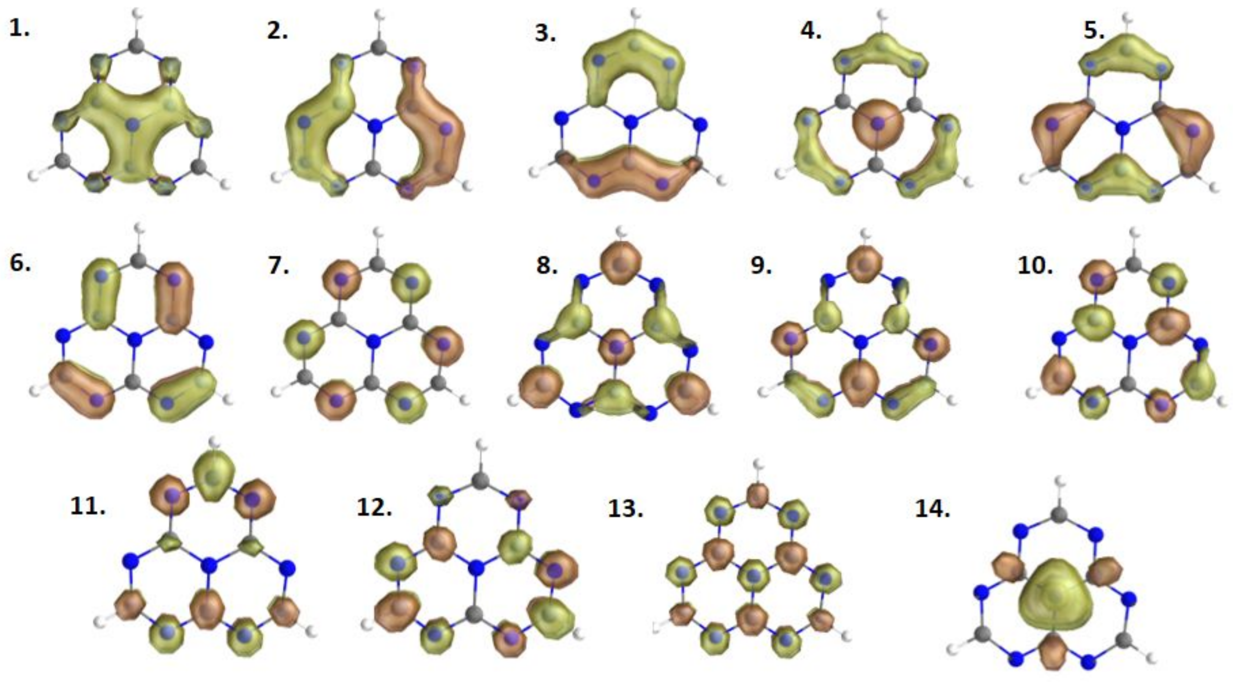

Figure S7. Pictures of orbital used in the active space for the RASSCF calculation of Azine-7N molecule.

Table S10. Orbital occupation numbers for S0, S1 and T1 RASSCF wave function of Azine-7N molecule

Symmetry	Orbital	Occupation (S_0)	Occupation (S_1)	Occupation (T_1)
a’’	1	1.983782	1.988205	1.986123
	2	1.973472	1.961174	1.962997
	3	1.973462	1.961162	1.962967

4	1.961335	1.962879	1.96331
5	1.957097	1.917353	1.923584
6	1.957085	1.91733	1.923564
7	1.931739	1.019098	1.00676
8	0.052456	0.982603	0.991391
9	0.056211	0.084941	0.08248
10	0.056221	0.084947	0.082521
11	0.034209	0.041309	0.039759
12	0.034193	0.041285	0.03956
13	0.024577	0.03236	0.029525
14	0.004161	0.005353	0.00546

XYZ structures

Azine

C 0.29571227656290 -1.11645908787096 -1.03842446284864
C -0.04706521800365 1.31934403450468 -0.99005292876944
C 0.44889438899612 -2.31754990910117 -0.37541655804453
N 0.08628397222642 0.05463651644450 1.07293826960345
C 0.24142901604711 -1.16577104466544 1.75136029428642
C 0.42220350495555 -2.34418800715984 1.00424489921486
C 0.21299932310255 -1.17410744943570 3.15795759373267
H 0.33577157985271 -2.12911359243048 3.64559726728640
C 0.03498007168762 -0.00829052945602 3.87548350920316
C -0.11764999391246 1.18868469660126 3.20489879248207
C -0.09441426872651 1.24350841420071 1.79929123508657
C -0.24866735229230 2.45439272405796 1.09960523157725
C -0.22511288640488 2.48968570312790 -0.27990354530738
H 0.01510339212988 -0.03257089837062 4.95677627128335

H	-0.25902244288657	2.12096931311574	3.73002904440926
C	0.11185538149163	0.08613576373209	-0.33136007490136
H	-0.34562607058744	3.42950164497546	-0.80191024024469
H	-0.38509300403014	3.34800643880998	1.68926923556455
H	-0.02330952982244	1.30634857072431	-2.06894641445060
H	0.31109702860637	-1.05509857845303	-2.11580715858094
H	0.58923666394547	-3.23310407603106	-0.93428102393420
H	0.53816416706202	-3.26338064732027	1.55789076335178

Azine-4N

C	0.03813971430702	-0.00056481045610	-0.02731061793232
C	0.07814549895228	-0.00075737330828	1.33440490689189
N	2.42649833736294	0.00180263335209	-0.01582022334103
C	2.35931375543170	0.00149434858365	1.38735964273490
C	3.61098072841515	0.00260507249302	2.05809915520899
H	3.59102787515874	0.00240648439895	3.13579786329187
C	4.77022905708588	0.00386876259749	1.34207367356935
C	3.67508554750766	0.00314365128744	-0.65950121016166
C	3.63033669954402	0.00335809888625	-2.07869051786668
C	2.43100686963253	0.00226702442213	-2.72518751660414
C	1.24494650783252	0.00076086547520	-0.77561606629751
H	2.38994097266008	0.00240549071682	-3.80656527520669
H	4.57372724444896	0.00438433799695	-2.60025499302293
H	-0.88495110793333	-0.00138266684719	-0.58404025101385
H	-0.83811581401694	-0.00176173924581	1.91014271217186

H	5.72735071490472	0.00471805534210	1.84707771671674
N	1.23139915998929	0.00096607757844	-2.09265265742376
N	4.82270416706622	0.00415082279864	-0.01273811490087
N	1.22537986165053	0.00026050392824	2.05805949318583

Azine-7N

C	0.16634198874869	-0.00030900832605	1.28293553976179
N	2.42647401719572	0.00164582945198	-0.01616256430193
C	2.42375059642879	0.00183715536339	1.38182284229140
C	4.68152689093320	0.00400098318993	1.29168668332194
C	3.63829182899181	0.00279849774858	-0.71280165894214
C	2.43152474226214	0.00110392059965	-2.62345306548530
C	1.21735194258382	0.00033204456802	-0.71750141597426
H	2.43360869268342	0.00111817391377	-3.70613279056932
H	-0.77253960503725	-0.00104729949036	1.82188726404947
H	5.61830371998505	0.00493042963289	1.83428486034754
N	4.78534345526285	0.00398610783490	-0.03393827110059
N	1.26257569449690	0.00084552112687	2.03631628489915
N	3.58238800443046	0.00302734641709	2.04081353724143
N	0.06768429131325	-0.00064903955422	-0.04307590992235
N	3.62935516709163	0.00261362992150	-2.04548494173326
N	1.23145334262951	-0.00000961239795	-2.05012519388357

M-18

C	-0.00000043743870	-0.70662045701795	-3.00815870400356

C	0.00000205444902	-1.17467622852689	-1.68213069071263
C	-0.00000118138438	0.00000338279323	-0.86991947110675
C	0.00000603341825	1.17468114874949	-1.68213738696087
C	0.00001206919335	0.70661768652952	-3.00816156298292
C	-0.00000787123210	-2.41154171064593	-1.00786318292512
C	-0.00001473847224	-1.22981246613527	1.15220915551105
C	-0.00000913622594	-2.41475754950239	0.36725448063764
C	0.00000676576646	-1.19885671736584	2.54575212786446
H	0.00000967641536	-2.14773032580089	3.06367400948466
C	0.00000065261126	-0.00000070880292	3.24651974894625
C	0.00000698219876	1.19885304477681	2.54575082743708
C	-0.00001551467705	1.22981560524123	1.15220850128550
C	-0.00000943955417	2.41475597794353	0.36725105586328
C	-0.00000687564534	2.41154663360946	-1.00786956349339
H	-0.00000758758623	3.34933834503220	-1.54895168118415
H	-0.00001450741059	3.34727964579075	0.91443493557583
H	-0.00000630821476	-3.34932823692033	-1.54895189304672
H	0.00001943917212	-1.32960282961593	-3.88994142792882
H	0.00001928736512	1.32959432586243	-3.88994622307423
H	-0.00001695381417	-3.34728339047063	0.91443511625796
H	0.00003216690395	0.00000151050256	4.32693334643853
H	0.00000749516649	2.14772173305753	3.06368212735406
N	-0.00001131100446	0.00000153091622	0.46980713476286

M-19

C	2.98363141253779	-0.67197232189630	0.00000239527147
C	1.64379523581690	-1.15110877999223	-0.00000012743472
C	0.85363129721870	0.01039615717304	-0.00000179191328
C	1.63173013360915	1.19106096766352	0.00000040901127
C	2.97637836409875	0.73122568506420	0.00000422239485
C	0.90903268615469	-2.35071488795533	-0.00000108443600
C	-1.14223788230075	-1.23964194016959	-0.00000607559390
C	-3.12714361704625	-0.10435904014374	0.00000137270410
C	-2.52772274998013	1.14529046266789	0.00000447386902
C	-1.13729411814747	1.23412947354281	-0.00000560771954
C	0.88776788120950	2.37879780436600	-0.00000331629722
H	1.36467935787788	3.35265415560339	-0.00000359912980
H	1.39893431759611	-3.31846730558397	0.00000311092889
H	3.87086737476372	-1.28607933858505	0.00000483588759
H	3.85585737937936	1.35603190257008	0.00000399305467
H	-4.20666611784659	-0.18061161151560	0.00000902081521
H	-3.10455077213565	2.05662011342767	0.00000863193398
N	-0.47860421273792	0.00301870900391	-0.00000453469492
N	-2.46679042413045	-1.27130541893933	-0.00000513537111
N	-0.44351573339630	2.39225984609004	-0.00000602015592
N	-0.41555807254103	-2.38241304239139	-0.00000517312464

M-20

C	2.98422675079318	-0.67770757724951	-0.00000420444143
C	1.64114012856340	-1.15087363251112	0.00000185205408

C	0.84649700186351	0.02033321817299	0.00000462839862
C	1.62776003735909	1.19218119831066	0.00000167794586
C	2.97495407614228	0.72156029792678	-0.00000176839727
C	0.92020565644086	-2.34441142498076	0.00000293967600
C	-1.13563715825302	-1.25654755535564	0.00000015549158
C	-3.08778309385414	-0.08801966215289	0.00001363810782
C	-1.21173333681789	1.18135289144322	0.00000119147697
C	0.92096775987644	2.40615401253159	0.00000085965667
H	1.43365692595256	3.35955671617733	-0.00000048264138
H	1.41417049970398	-3.30980602441596	0.00000302282842
H	3.86914019766972	-1.29477797173033	-0.00000541511950
H	3.85450968751445	1.34691026053815	-0.00000496401160
H	-4.16933236952160	-0.12465074330290	-0.00002313610735
N	-0.48692967794005	-0.00936660040390	0.00000344109261
N	-2.46777956096497	-1.27038959400646	0.00000091474798
N	-0.41561791085192	-2.38725186578453	0.00000009539302
C	-0.46330011467843	2.38052132794215	0.00000145587433
N	-2.54654047501853	1.12991948034776	0.00000185821560
H	-1.04248017397894	3.29154846850337	0.00000223975896

M-21

C	2.98754079831770	-0.66532314629152	0.00000061867273
C	1.64569499032377	-1.15822383945631	0.00000074271264
C	0.84946676749080	0.00016284124775	0.00000004301845
C	1.60895948239558	1.18293399525891	0.00000087339543

C	2.96560900617353	0.73238477730920	-0.00000279427711
C	0.91893335287030	-2.35468557353108	0.00000095357136
C	-1.13404737785230	-1.25828524049334	-0.00000184072971
C	-3.05938598157385	-0.06115380008536	-0.00000127681192
C	-1.17256558640588	1.19576810814606	0.00000132487876
C	0.84508525409068	2.35603820777391	0.00000116096124
H	1.30501376848568	3.33793190722936	0.00000094887164
H	1.40943006365319	-3.32164448167151	0.00000522254578
H	3.88021336043024	-1.27096320148972	-0.00000070532990
H	3.83885258218180	1.36572085967354	-0.00000481103286
H	-4.14123244869072	-0.07811621437101	-0.00000298578183
N	-0.48041203791834	-0.02074818823889	-0.00000047676471
N	-2.46507484160379	-1.25674051483676	-0.00000470834731
N	-0.41489290983253	-2.39321299110535	0.00000004068791
N	-2.50285308816848	1.15250476510341	0.00000529785131
N	-0.48926236436738	2.35263384982872	0.00000237190810
